# Use of 2-hourly creatinine clearance to inform cessation of renal replacement therapy

**DOI:** 10.1186/cc9550

**Published:** 2011-03-11

**Authors:** O Solymos, S Frohlich, N Conlon

**Affiliations:** 1 St Vincent's University Hospital, Dublin, Ireland; 2 St James's Hospital, Dublin, Ireland

## Introduction

Acute kidney injury (AKI) is a common problem in critically ill patients, with a reported incidence of 1 to 25% and a poor prognosis. Although optimal dosing of renal replacement therapy (RRT) is relatively well understood, appropriate timing of commencing and ceasing RRT in patients with AKI has been under debate for a long time. From the viewpoint of an early renal support strategy, the goal of early RRT is to maintain solute clearance and fluid balance to prevent subsequent multiorgan damage, while waiting for the recovery of renal function. It has previously been noted that 2-hourly creatinine clearance accurately reflects the more cumbersome 24-hour value [1]. The aim of the present study was to evaluate whether routine measurement of creatinine clearance (CrCl) could help to predict when to cease dialysis, and determine what value for CrCl best predicted remaining dialysis-free in critically ill patients receiving CRRT.

## Methods

Two-hourly creatinine clearance is calculated daily on most patients on CRRT in our ICU. If CrCl is greater than 20 ml/minute, CRRT is ceased. Our retrospective chart review examined records for all patients admitted to our ICU in 2008 and determined whether a CrCl greater than 20 ml/minute accurately predicted remaining dialysis-free 5 days later.

## Results

Forty-one patients were suitable for analysis. Of these, 12 (30%) never reached CrCl >20 ml/minute and remained on dialysis leaving the ICU. Of the remaining 29 patients, in 23 (79%) having a CrCl >20 ml/minute meant they remained dialysis-free for at least the following 5 days. Six patients (21%), despite having a CrCl >20 ml/minute, resumed dialysis within 5 days for metabolic or fluid-removal reasons.

## Conclusions

Although this is a small retrospective study it suggests that 2-hourly creatinine clearance values may accurately predict when CRRT should be discontinued. These pilot results should be used to inform a larger prospective study.
